# Development and Evaluation of a Robotic System for Safe Cardiac Sheath Delivery

**DOI:** 10.1109/TBME.2025.3553014

**Published:** 2025-09-01

**Authors:** Weizhao Wang, Carlo Saija, Zhouyang Xu, Aya Zeidan, Joshua Wilcox, Tiffany Patterson, Simon Redwood, Shuangyi Wang, Kawal Rhode, Richard Housden

**Affiliations:** School of Biomedical Engineering and Imaging Sciences, King’s College London, WC2R 2LS London, U.K.; School of Biomedical Engineering and Imaging Sciences, King’s College London, WC2R 2LS London, U.K.; School of Biomedical Engineering and Imaging Sciences, King’s College London, WC2R 2LS London, U.K.; School of Biomedical Engineering and Imaging Sciences, King’s College London, WC2R 2LS London, U.K.; Cardiovascular Department, Guy’s and St Thomas’ NHS Foundation Trust, U.K.; Cardiovascular Department, Guy’s and St Thomas’ NHS Foundation Trust, U.K.; School of Cardiovascular Medicine and Sciences, King’s College London, WC2R 2LS London, U.K.; State Key Laboratory of Multimodal Artificial Intelligence Systems, Institute of Automation, Chinese Academy of Sciences, China, also with the School of Artificial Intelligence, University of Chinese Academy of Sciences, China, and also with the Centre for Artificial Intelligence and Robotics, Hong Kong Institute of Science & Innovation, Chinese Academy of Sciences, China; School of Biomedical Engineering and Imaging Sciences, King’s College London, WC2R 2LS London, U.K.; School of Biomedical Engineering and Imaging Sciences, King’s College London, WC2R 2LS London, U.K.

**Keywords:** Medical robots and systems, modeling and control for soft robots, cardiac guide sheath, safe delivery, tissue contact reduction

## Abstract

**Objective:**

This study aimed to develop and evaluate a 3-degree-of-freedom (DoF) robotic system for the safe delivery of cardiac sheaths through challenging anatomical structures, including the fossa ovalis and pathways with tight curves.

**Methods:**

The robot and its kinematic model were built on a previously proposed single-DoF actuation module and bending model. A sheath delivery strategy (SDS) was developed, combining two control methods: tip position control to approach an optimal entry point and point-constrained control to maintain consistent navigation through this point, minimizing tissue contact. Technical performance was evaluated through trajectory-following and point-crossing tests, followed by feasibility experiments in a simulated scenario. Trials were conducted by three cardiologists using a validated phantom model under fluoroscopic guidance, comparing SDS with joint control (JC) and manual control (MC).

**Results:**

Average root mean square errors were 2.10 mm for tip position control and 1.86 mm for point-constrained control. SDS outperformed MC with significantly shorter trajectory lengths and lower root mean square jerk. Compared to JC, SDS reduced sheath-induced movements (an indirect measure of force) and increased retraction success rates at the fossa ovalis.

**Conclusion:**

The proposed robotic system reduced tissue wall contact compared to JC and provided smoother, more controlled operations than MC, ensuring safer and more effective delivery through confined pathways.

**Significance:**

This work contributes to advancing robotic-assisted cardiac sheath delivery, providing a reliable and safer method for navigating challenging anatomical structures.

## I Introduction

**C**ARDIAC interventions require skilled catheter manipulation to deliver devices to targets like valves and coronary arteries [1]. Challenges include navigating through complex vascular anatomy, such as the approximately 90° angle between the tricuspid valve (TV) and inferior vena cava (IVC), and multiple curves when accessing the mitral valve (MV) through the septum [2]. Meanwhile, these catheters, designed for flexibility in tortuous anatomy [3], are difficult to position precisely due to blood flow disturbances and lack of support [4]. Clinically, commercial steerable guide sheaths with enhanced stiffness and bendable tips are commonly employed to not only create a stable pathway for the delivery but also augment flexibility and workspace for the positioning of the other catheters [5]. Examples include the Agilis steerable sheath from St Jude Medical (St. Paul, MN, USA) used in ablation procedures [6], [7], [8], the Heli-FX EndoAnchor steerable sheath from Medtronic (Minneapolis, MN, USA) for endovascular interventions [9], [10], and the FlexCath steerable sheath from Medtronic (Minneapolis, MN, USA) for valve procedures [11].

The sheath’s structure is simple, typically featuring a planar bendable section (BS) at the tip and a knob on the handle for its flexion adjustment. Cardiologists manipulate it using the thumb and index fingers for tip bending, while the remaining fingers and palm stabilize the handle, enabling translational and rotational movements. However, its inherent stiffness and tip spatial movement challenge the manipulation [12], [13], and safe delivery depends on the cardiologist’s experience and skill [14], [15]. Robotic operation can improve precision and dexterity, eliminate physiological tremors, reduce operator fatigue, and mitigate occupational hazards like radiation exposure and degenerative joint disease from wearing lead aprons [16], [17]. In robotic surgery, the primary domains of study are navigation and control. The integration of imaging modalities such as fluoroscopy and transesophageal echocardiography (TEE) enables accurate localization of both tissue and robotic instruments. This paper focuses on the other part: control algorithms for modulating the robot’s movements in response to localization feedback. Currently, Sensei and Magellan (Auris Surgical Robotics, Silicon Valley, CA, USA) assist cardiac procedures by navigating balloons, stents, and other materials through steerable sheaths [18], [19]. Systems like Amigo (Catheter Precision, Mount Olive, NJ, USA) and Niobe (Stereotaxis, St. Louis, MO, USA) offer robotic guide catheters for tasks like radiofrequency ablation and cardiac mapping. These systems feature teleoperation workstations for remote control. However, to establish a delivery pathway in narrow spaces without tissue contact, BS shape adjustment and tip positioning are crucial, and these require a precise model and control strategies of state (pose and/or shape).

Many research groups are either focusing on cardiac catheters or in a more general context of continuum robots. Ouyang [20] proposed a shape control method of a multisection continuum robot based on the Jacobian matrix by controlling several reference points of each section on the robot to reach the desired positions. Almanzor [21] presented a novel image-based deep learning approach for closed-loop kinematic shape control of soft continuum robots with a user-friendly way to control the robot’s 3D shape and configuration through teleoperation using only 2D hand-drawn images of the desired target state. Li [22] proposed an optimization method based on null space to reduce the length difference among the driving tendons to avoid the over-deformed configurations of the continuum robot. However, these methods are specifically for multi-section or redundant continuum robots and can hardly be implemented in a steerable guide sheath which commonly only has one degree of freedom (DoF) for bending.

**Fig. 1:**
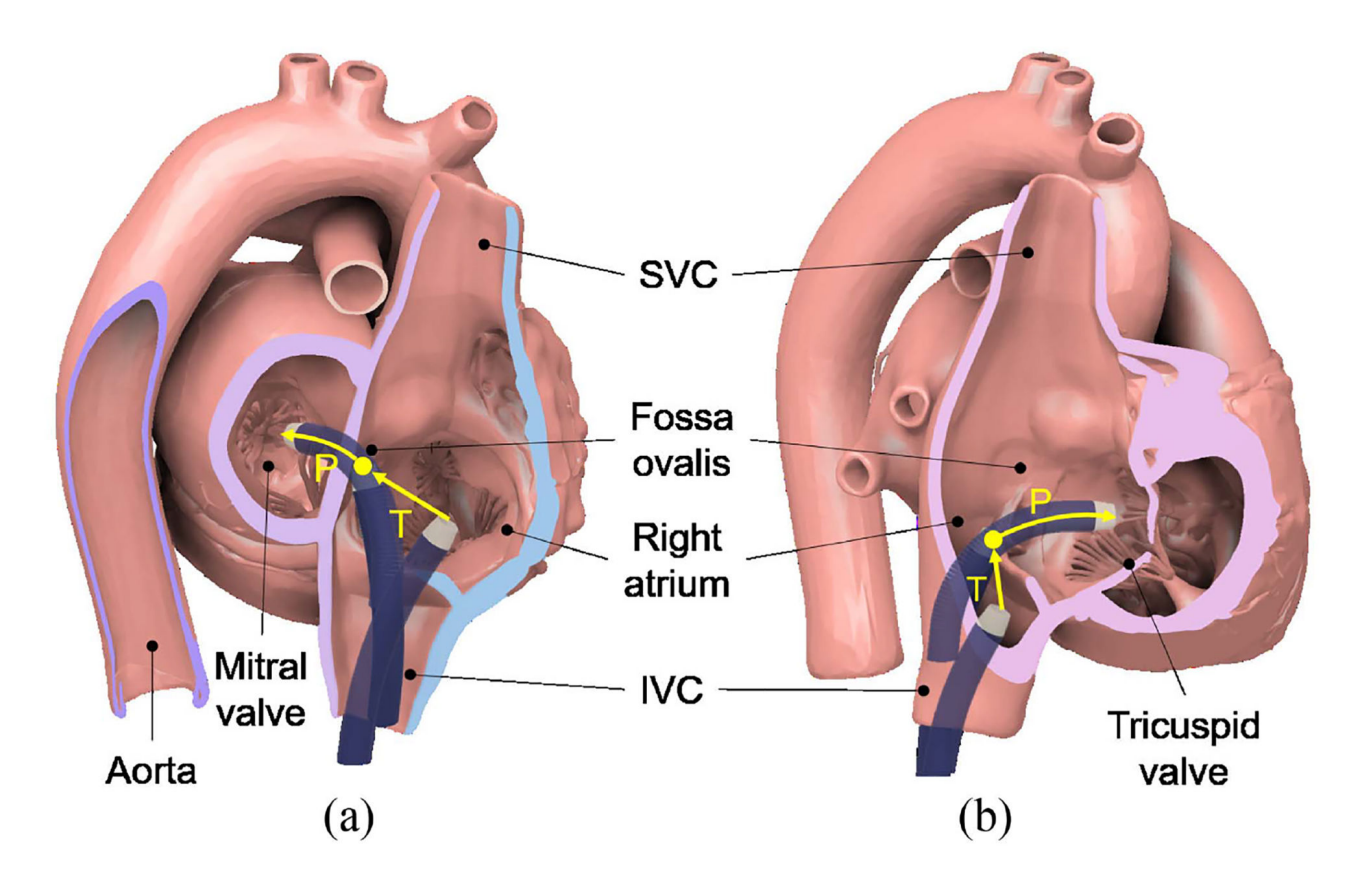
A safe delivery strategy combines tip position control (”T”) and point-constrained control (”P”). (a) Accessing the MV, the tip moves from the IVC to the fossa ovalis (yellow dot) and crosses it. (b) Accessing the TV, the tip moves from the IVC to the Eustachian ridge (yellow dot) and crosses it.

Building on the single-DoF actuation module and bending model from previous work [23], we developed a 3-DoF robotic system by integrating translational mechanisms and establishing a full kinematic model that precisely relates motor movements to the tip’s state. Furthermore, we propose a safe sheath delivery strategy (SDS) (Fig. 1) with two control methods: tip position control (”T” in the figure), which employs differential kinematics for precise navigation around obstacles to approach an optimal entry point (yellow dots), and point-constrained control (”P” in the figure), which fixes the tip’s current position as a reference point to ensure consistent passage through this point, thereby minimizing tissue contact during delivery. Illustrative examples in Fig. 1 include manoeuvring through the fossa ovalis, and negotiating paths with tight curves between the TV and the IVC. Further details are provided in Section II. In Section III, we fabricate the robotic system and evaluate its control performance through tip trajectory tracking and point-constrained delivery experiments using two sizes of sheaths. Tip following experiments include three scenarios: pre-defined trajectory following, master following in simulated fluoroscopy views, and master following in 3D space. Point-constrained control is evaluated in two scenarios: advancing and retracting. Subsequent feasibility experiments in a simulated scenario were conducted by three cardiologists using a validated phantom model under fluoroscopic guidance, comparing SDS with joint control (JC) and manual control (MC) for tasks involving crossing the fossa ovalis and approaching the tricuspid annulus. Results are presented and discussed in Section IV. Finally, Section V summarizes the conclusions and outlines future research directions.

In summary, the major contributions are as follows: 1) We developed a 3-DoF robotic system for remote manipulation of commercial sheaths, incorporating an SDS method with tip position control for precise entry point navigation and point-constrained control for shape-constrained crossing; 2) We conducted feasibility experiments with cardiologists using a phantom model under fluoroscopic guidance, demonstrating reduced tissue wall contact compared to JC and smoother, more controlled operations than MC, ensuring safer and more effective delivery through confined pathways.

## II Methods

### A Robotic System Design

**Fig. 2:**
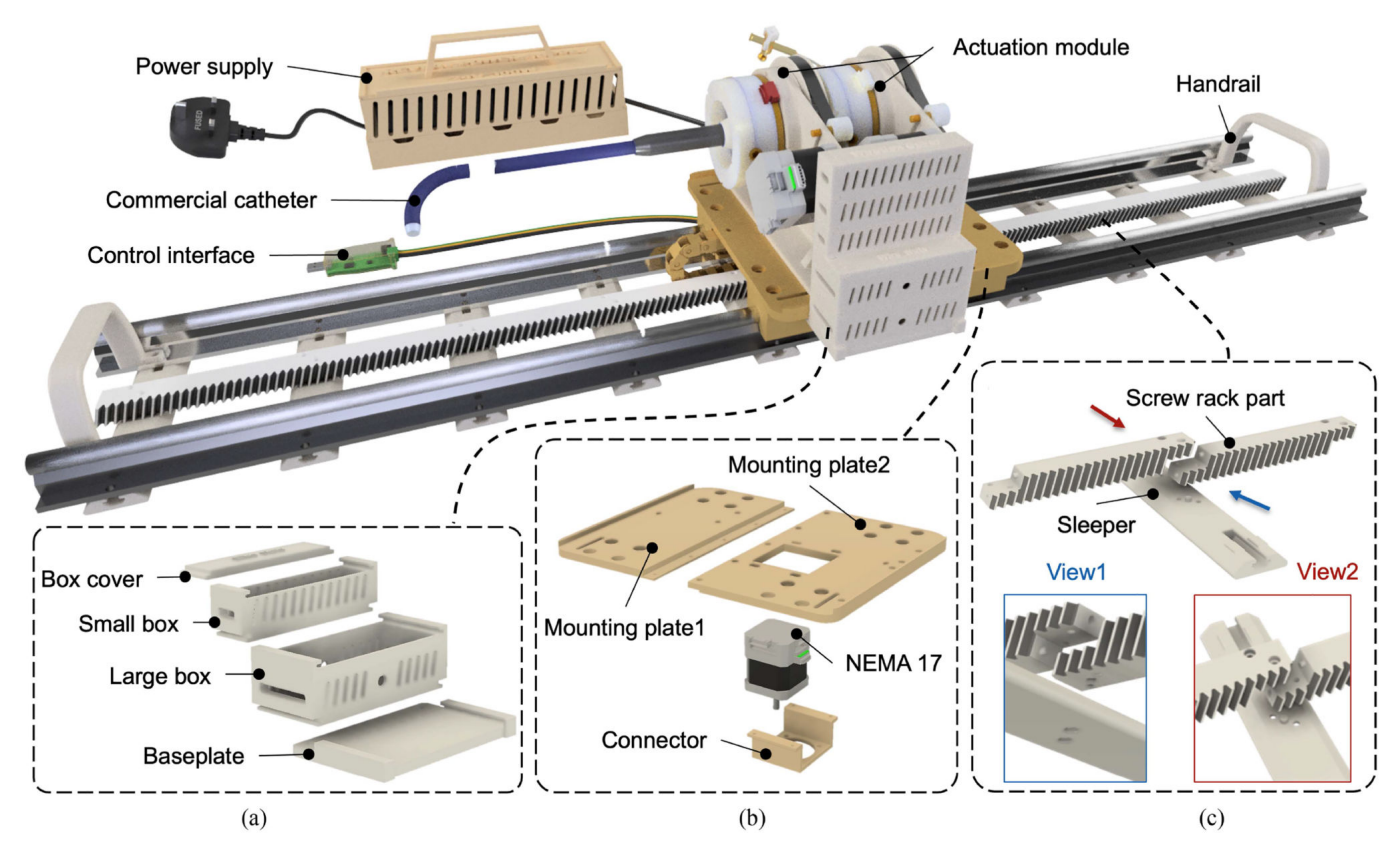
Description of the robotic system developed based on previously designed actuation modules: (a) Stackable boxes are assembled on the side of the mounting plate to store PCBs. (b) Mounting plates are connected with linear bearings and are driven through pinion and rack mechanisms. (c) The rack is divided into small segments, each 100 mm in length, to enhance the quality of 3D printing.

A robotic system consisting of transmission and housing mechanisms was designed to translate, rotate, and bend guide sheaths featuring one BS at the tip. Fig. 2 shows the manipulation of a guide sheath (760 mm length, 30 Fr outer diameter) from Edwards Lifesciences (Irvine, CA, USA).

#### 1) Transmission Mechanism

The sheath has three DoFs: translation, rotation, and bending. The rotation and bending DoFs are achieved using two actuation modules from prior work [23], where one module clamps the knob and the other clamps the handle. Tip bending is controlled by turning the knob, while combined turning of both the handle and knob enables tip rotation, with knob turning compensating as needed. The translational movement is achieved through a helical pinion gear (Module 1.5, 20 teeth, 32 mm pitch diameter) and gear rack (Module 1.5, 14 mm face width, 950 mm length) mechanism, driven by a NEMA 17 motor (0.4 Nm torque, 1.8° step angle, 42.3 × 42.3 mm^2^ frame, 5 mm shaft) attached at the base of mounting plates via a specialized connector, as detailed in Fig. 2(b). The mounting plates are assembled with four linear rail bearings (SBR12UU), allowing relative linear movement along two rail guide shafts (SBR12, 1000 mm length). Importantly, the pinion and rack mechanism enables future integration of mechanisms for manipulating other catheters. For optimized printing quality, the mounting plate was segmented into two separate plates, and the gear rack was divided into sections with a length of 100 mm. Views 1 and 2 of Fig. 2(c) depict the assembly of two sections and a sleeper unit, securely fastened using dowel pins and sockets. The overall robot footprint measures 985 × 246 × 174 mm^3^. The motion ranges for each joint are as follows: 0 to 320 mm for translation, *−*360° to 360° for rotation, and 0° to 70° for bending.

#### 2) Housing Mechanism

The stackable collection boxes (Large: 90 × 38 × 30 mm^3^; Small: 90 × 20 × 24 mm^3^), depicted in Fig. 2(a), are designed with slots (3 × 2 mm^2^) and interlocking edges for efficient storage of multiple CAN bus converters and cables. A specialized drag chain (15 mm width) addresses wiring requirements, simplifying cable management. The stackable collection boxes and the drag chain box are 3D printed using PLA material. The platform’s portability is enhanced by incorporating dual handrails on both sides.

#### 3) Control Interface

A PC running Ubuntu 20.04 serves as the main controller, which processes input commands, executes control algorithms, and communicates with the actuation system via a CAN bus at a 250 Kbps baud rate. The entire system is powered by a 24 V, 300 W, 12.5 A DC adapter. A user interface (UI) provides real-time status updates and visualizes the main controller’s functions. Additional details are provided in Section III-A.

### B Kinematic Modeling

**Fig. 3:**
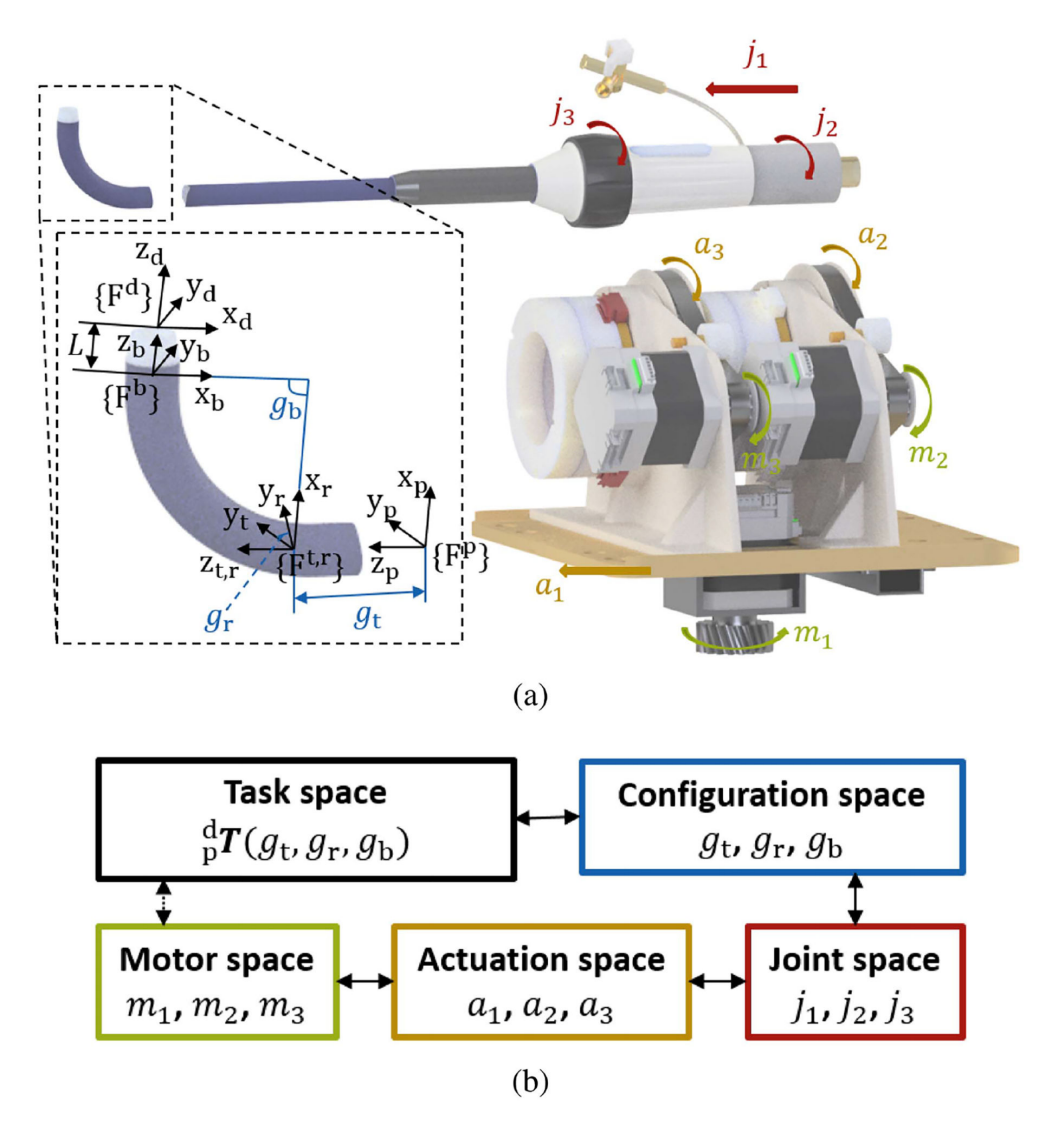
Definition of kinematic spaces: (a) Explanation of coordinate frames and model variables. (b) Mapping chain from motor space to task space.

The robotic system requires a reliable model to provide a precise motion relationship between the motors and the sheath tip for optimal control performance. Fig. 3(a) shows the definition of coordinate frames and each variable of the model. To enhance clarity, this paper uses bold black letters for vectors and matrices, italic letters for variables, and roman style for non-variable labels. In task space, two coordinate frames ({F^p^} and {F^d^}) are attached to the proximal and distal ends of the tip, respectively, with the tip’s pose ${\;}_{\text{p}}^{d}T(4\times 4\;\text{matrix})$ represented in {F^d^} relative to {F^p^}. The tip is characterised by three configuration parameters: the translation distance along the z-axis (*g*_t_), the rotation angle about the z-axis (*g*_r_), and the planar bending angle about the y-axis (*g*_b_). The manipulation parameters in joint space are the translation distance *j*_1_, rotation angle *j*_2_ of the handle, and rotation angle *j*_3_ of the knob, collectively influencing the pose and shape of the tip. The system has three motors (*m*_1_, *m*_2_, and *m*_3_) for the actuation of the plate (*a*_1_) and two modules (*a*_2_ and *a*_3_). To simplify the modeling process, the relationship is decomposed into a mapping chain, as illustrated in Fig. 3(b), which translates motor space coordinates to the task space pose of the tip. The variables in each space are marked in different colors.

#### 1) Mapping From Configuration to Task Space

To establish the correlation between the configuration parameters and the tip’s pose, three additional frames ({F^t^}, {F^r^}, and {F^b^}) are attached to the translation, rotation, and bending joints, and introduced to facilitate the transfer of parameter influences onto the tip’s pose. *L* represents the length of the straight tip. Then the mapping can be calculated as:

(1) $${\;}_{\text{p}}^{d}T\left(g_{\text{t}},g_{\text{r}},g_{\text{b}}\right){=}_{\text{p}}^{t}T{\left(g_{\text{t}}\right)}_{\text{t}}^{r}T{\left(g_{\text{r}}\right)}_{\text{r}}^{b}T{\left(g_{\text{b}}\right)}_{\text{b}}^{d}T$$

Based on the planar bendable tip position model (*x*_p_(*g*_b_), *y*_p_(*g*_b_)) from prior work [23], transformation ${\;}_{\text{r}}^{b}T\left(g_{\text{b}}\right)$ can be written as follow:

(2) $${\;}_{\text{r}}^{b}T\left(g_{\text{b}}\right)=\left[\begin{array}{cccc} \text{c}g_{\text{b}} & 0 & \text{s}g_{\text{b}} & x_{\text{p}}\left(g_{\text{b}}\right)\\ 0 & 1 & 0 & 0\\ -\text{s}g_{\text{b}} & 0 & \text{c}g_{\text{b}} & y_{\text{p}}\left(g_{\text{b}}\right)\\ 0 & 0 & 0 & 1 \end{array}\right]$$

where s∗ and c∗ are the sine and cosine of angle (*∗*). Then ${\;}_{\text{p}}^{d}T\left(g_{\text{t}},g_{\text{r}},g_{\text{b}}\right)$can be calculated as (3).

(3) $${\;}_{\text{p}}^{d}T=\left[\begin{array}{cccc} \text{c}g_{\text{r}}\text{c}g_{\text{b}} & -\text{s}g_{\text{r}} & \text{c}g_{\text{r}}\text{s}g_{\text{b}} & x_{\text{p}}\left(g_{\text{b}}\right)\text{c}g_{\text{r}}+L\text{c}g_{\text{r}}\text{s}g_{\text{b}}\\ \text{s}g_{\text{r}}\text{c}g_{\text{b}} & \text{c}g_{\text{r}} & \text{s}g_{\text{r}}\text{s}g_{\text{b}} & x_{\text{p}}\left(g_{\text{b}}\right)\text{s}g_{\text{r}}+L\text{s}g_{\text{r}}\text{s}g_{\text{b}}\\ -\text{s}g_{\text{b}} & 0 & \text{c}g_{\text{b}} & g_{\text{t}}+y_{\text{p}}\left(g_{\text{b}}\right)+L\text{c}g_{\text{b}}\\ 0 & 0 & 0 & 1 \end{array}\right]$$

#### 2) Mapping From Joint to Configuration Space

The mapping from *j*_1_ and *j*_2_ to *g*_t_ and *g*_r_ are straightforward and linear due to the direct correlation between the movements of the tip and the manipulation of the sheath handle, with a 2 × 2 identity matrix ***I***_2_ incorporated. Based on the study of the correlation between *j*_3_ and *g*_b_ from prior work [23], a constant value *C*_dz_ aptly models the dead zone in the initial phase, addressing the lack of tip bending during knob rotation. Subsequently, a first-order polynomial fitting (*C*_p1_, *C*_p2_) is employed for the following phase. *δC* is an additional input compensator. *C*_j_ equals to (*C*_dz_ − *C*_p2_)/(*C*_p1_ ± *δC*). The mapping can be calculated as follows:

(4) $$\begin{array}{l} \left[\begin{array}{l} g_{\text{t}}\\ g_{\text{r}}\\ g_{\text{b}} \end{array}\right]=\left[\begin{array}{cc} I_{2} & 0_{2\times 1}\\ 0_{1\times 2} & \{\begin{array}{cc} C_{dz}/j_{3}, & \;\text{if}\;0\leq j_{3}<C_{\text{j}}\\ \left(C_{\text{p}1}\pm \delta C\right)+C_{\text{p}2}/j_{3}, & \;\text{if}\;C_{\text{j}}\leq j_{3} \end{array} \end{array}\right]\\ \;\times \left[\begin{array}{l} j_{1}\\ j_{2}\\ j_{3} \end{array}\right] \end{array}$$

#### 3) Mapping From Actuation to Joint Space

The handle rotation is accomplished through the simultaneous rotation of two chucks, denoted as *a*_2_ and *a*_3_. Meanwhile, the knob rotation and handle translation are achieved by rotating the forward chuck (*a*_3_) and translating the mounting plates (*a*_1_). The mapping can be calculated as follows:

(5) $$\left[\begin{array}{l} j_{1}\\ j_{2}\\ j_{3} \end{array}\right]=\left[\begin{array}{ccc} 1 & 0 & 0\\ 0 & 1 & 0\\ 0 & -1 & 1 \end{array}\right]\left[\begin{array}{l} a_{1}\\ a_{2}\\ a_{3} \end{array}\right]$$

#### 4) Mapping From Motor to Actuation Space

This mapping is specifically designed for the transformation of transmission ratios in mechanisms, with the transmission ratios denoted as *M*_1_ for pinion and rack mechanisms and *M*_2_ for pulley belt mechanisms, as indicated in (6) below.

(6) $$\left[\begin{array}{l} a_{1}\\ a_{2}\\ a_{3} \end{array}\right]=\left[\begin{array}{ccc} M_{1} & 0 & 0\\ 0 & M_{2} & 0\\ 0 & 0 & M_{2} \end{array}\right]\left[\begin{array}{l} m_{1}\\ m_{2}\\ m_{3} \end{array}\right]$$

By combining equations from (3) to (6), we can get the mapping (7) between motor space coordinates and task space pose ${\;}_{\text{p}}^{d}T$ of the tip. The position ***P***_3×1_ of the tip can be expanded as (8), shown at the bottom of this page.

(7) $${\;}_{\text{p}}^{d}T=\left[\begin{array}{cc} R_{3\times 3} & P_{3\times 1}\\ 0_{1\times 3} & 1 \end{array}\right]$$

### C Tip Position Control

Position controllers often rely on the inverse kinematic model, which requires the inverse Jacobian matrix. Initially, the Jacobian matrix in task space *J* was calculated based on (8), as shown in (9) and (10).

(8) $${\dot{P}}_{3\times 1}=J\dot{M}$$

(9) $$P_{3\times 1}=\left[\begin{array}{c} P_{1}\\ P_{2}\\ P_{3} \end{array}\right]=\left[\begin{array}{c} x_{\text{p}}\left(C_{\text{p}1}M_{2}\left(m_{3}-m_{2}\right)+C_{\text{p}2}\right)\text{c}\left(M_{2}m_{2}\right)+L\text{c}\left(M_{2}m_{2}\right)\text{s}\left(C_{\text{p}1}M_{2}\left(m_{3}-m_{2}\right)+C_{\text{p}2}\right)\\ x_{\text{p}}\left(C_{\text{p}1}M_{2}\left(m_{3}-m_{2}\right)+C_{\text{p}2}\right)\text{s}\left(M_{2}m_{2}\right)+L\text{s}\left(M_{2}m_{2}\right)\text{s}\left(C_{\text{p}1}M_{2}\left(m_{3}-m_{2}\right)+C_{\text{p}2}\right)\\ M_{1}m_{1}+y_{\text{p}}\left(C_{\text{p}1}M_{2}\left(m_{3}-m_{2}\right)+C_{\text{p}2}\right)+L\text{c}\left(C_{\text{p}1}M_{2}\left(m_{3}-m_{2}\right)+C_{\text{p}2}\right) \end{array}\right]$$

(10) $$J=\left[\begin{array}{ccc} 0 & \frac{\partial P_{1}}{\partial m_{2}} & \frac{\partial P_{1}}{\partial m_{3}}\\ 0 & \frac{\partial P_{2}}{\partial m_{2}} & \frac{\partial P_{2}}{\partial m_{3}}\\ M_{1} & \frac{\partial P_{3}}{\partial m_{2}} & \frac{\partial P_{3}}{\partial m_{3}} \end{array}\right]$$

**Fig. 4:**
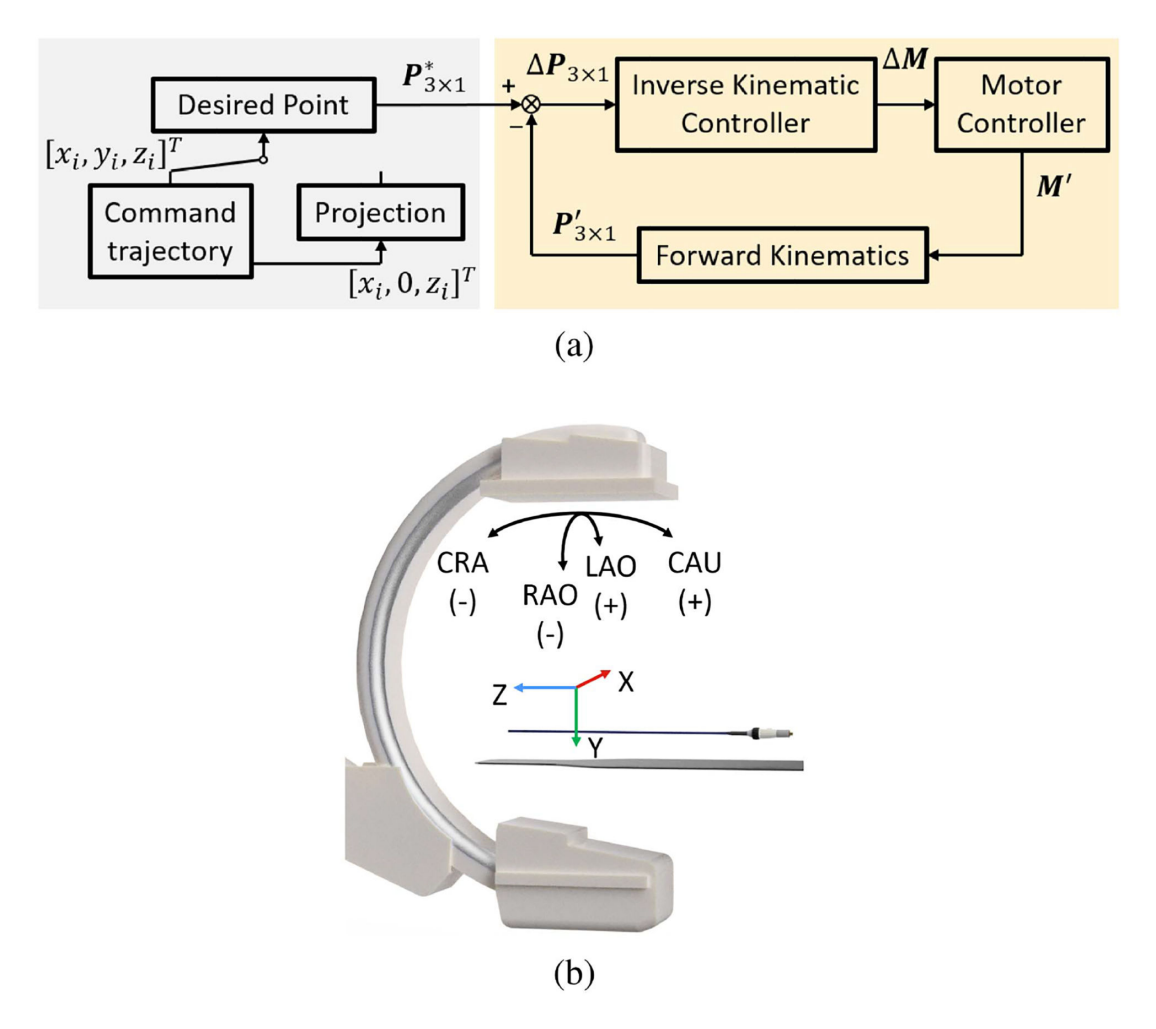
Tip position control in free space and under fluoroscopy guidance: (a) Workflow for 2D/3D tip position control. (b) Registration process between the C-arm and the sheath.

In medical robotic systems, the command trajectory$\cup_{i=1}^{n}(x(i),y(i),z(i))$ can be generated either through a master controller or defined before the procedure. Master-slave control enables clinicians to operate a robot remotely, mitigating radiation exposure risks. In addition, the trajectory can also be defined and updated to facilitate autonomous task manipulation. The control workflow, illustrated in Fig. 4(a), involves acquiring a desired point $\left(P_{3\times 1}^{⋆}\right)$ from the command trajectory during each control cycle. Subsequently, the position difference (Δ***P***_3×1_) is adjusted based on the real-time tip position $\left(P_{3\times 1}^{\prime }\right)$ calculated using (8). Finally, the necessary motor movements (Δ ***M***) are determined through the inverse Jacobian matrices. This integrated process allows for positioning the catheter tip in real-time. Notably, some procedures are mainly guided by fluoroscopy which provides a 2D view. In such cases, the 2D command trajectory $\cup_{i=1}^{n}(x(i),y(i),z(i))$ is projected via LAO-RAO and CAU-CRA angles (*θ*_LR_ and *θ*_UR_). The coordinate system is defined in Fig. 4(b). Consequently, the desired point is obtained using (11).

(11) $$\begin{array}{l} P_{3\times 1}^{⋆}=\left[\begin{array}{ccc} \text{c}\theta_{\text{LR}} & -\text{s}\theta_{\text{LR}} & 0\\ \text{s}\theta_{\text{LR}} & \text{c}\theta_{\text{LR}} & 0\\ 0 & 0 & 1 \end{array}\right]\left[\begin{array}{ccc} 1 & 0 & 0\\ 0 & \text{c}\theta_{\text{UR}} & -\text{s}\theta_{\text{UR}}\\ 0 & \text{s}\theta_{\text{UR}} & \text{c}\theta_{\text{UR}} \end{array}\right]\left[\begin{array}{c} x(i)\\ 0\\ z(i) \end{array}\right]\\ \;=\left[\begin{array}{c} x(i)\text{c}\theta_{\text{LR}}+z(i)\text{s}\theta_{\text{LR}}\text{s}\theta_{\text{UR}}\\ x(i)\text{s}\theta_{\text{LR}}-z(i)\text{c}\theta_{\text{LR}}\text{s}\theta_{\text{UR}}\\ z(i)\text{c}\theta_{\text{UR}} \end{array}\right] \end{array}$$

### D Point-Constrained Control

Based on the BS shape model (*x*_p_(λ_B_, *g*_b_), *y*_p_(λ_B_, *g*_b_)) from prior work, the shape of the BS in 3D space is calculated as (12), accounting for translation and rotation movements (*g*_t_ and *g*_r_). Here, λ_B_ ranging from 0 to *g*_b_ represents the curvature along the BS and *G*_B_ = (λ_B_, *g*_t_, *g*_r_, *g*_b_) are the input variables. Additionally, the shape of the straight section (SS) with a length of *L* is determined by (13), where λ_S_ ranging from 0 to *L* represents the length along the SS. *G*_S_ = (λ_S_, *g*_t_, *g*_r_, *g*_b_) are the input variables.

(12) $$S_{\text{B}}\left(G_{\text{B}}\right)=\left[\begin{array}{c} X_{\text{B}}\\ Y_{\text{B}}\\ Z_{\text{B}} \end{array}\right]=\left[\begin{array}{c} x_{\text{p}}\left(\lambda_{\text{B}},g_{\text{b}}\right)cg_{\text{r}}\\ x_{\text{p}}\left(\lambda_{\text{B}},g_{\text{b}}\right)sg_{\text{r}}\\ y_{\text{p}}\left(\lambda_{\text{B}},g_{\text{b}}\right)+g_{\text{t}} \end{array}\right]$$

(13) $$S_{\text{S}}\left(G_{\text{S}}\right)=\left[\begin{array}{c} X_{\text{S}}\\ Y_{\text{S}}\\ Z_{\text{S}} \end{array}\right]=\left[\begin{array}{l} \left(x_{\text{p}}\left(g_{\text{b}},g_{\text{b}}\right)+\text{s}g_{\text{b}}\lambda_{\text{S}}\right)\text{c}g_{\text{r}}\\ \left(x_{\text{p}}\left(g_{\text{b}},g_{\text{b}}\right)+\text{s}g_{\text{b}}\lambda_{\text{S}}\right)\text{s}g_{\text{r}}\\ y_{\text{p}}\left(g_{\text{b}},g_{\text{b}}\right)+\text{c}g_{\text{b}}\lambda_{\text{S}}+g_{\text{t}} \end{array}\right]$$

The planar bending nature allows for the decoupling of tip rotation by rotating frame {F^p^} about its z-axis with an angle of *−g*_r_. Subsequently, the shape function (12) is computed relative to the new frame $\left\{{\bar{\text{F}}}^{\text{p}}\right\}$ using the formulation in (14). In this frame, ${\bar{Y}}_{\text{B}}\;$ or ${\bar{Y}}_{\text{S}}\;$ equals zero. Similarly, ${\bar{S}}_{\text{S}}$ can be computed using analogous procedures, as shown in (15).

(14) $$\begin{array}{l} {\bar{S}}_{\text{B}}\left(G_{\text{B}}\right)=\left[\begin{array}{ccc} \text{c}\left(-g_{\text{r}}\right) & -\text{s}\left(-g_{\text{r}}\right) & 0\\ \text{s}\left(-g_{\text{r}}\right) & \text{c}\left(-g_{\text{r}}\right) & 0\\ 0 & 0 & 1 \end{array}\right]S_{\text{B}}\left(G_{\text{B}}\right)\\ \;=\left[\begin{array}{c} x_{\text{p}}\left(\lambda_{\text{B}},g_{\text{b}}\right)\\ 0\\ y_{\text{p}}\left(\lambda_{\text{B}},g_{\text{b}}\right)+g_{\text{t}} \end{array}\right] \end{array}$$

(15) $${\bar{S}}_{\text{S}}\left(G_{\text{S}}\right)=\left[\begin{array}{c} x_{\text{p}}\left(g_{\text{b}},g_{\text{b}}\right)+\text{s}g_{\text{b}}\lambda_{\text{S}}\\ 0\\ y_{\text{p}}\left(g_{\text{b}},g_{\text{b}}\right)+\text{c}g_{\text{b}}\lambda_{\text{S}}+g_{\text{t}} \end{array}\right]$$

**Fig. 5:**
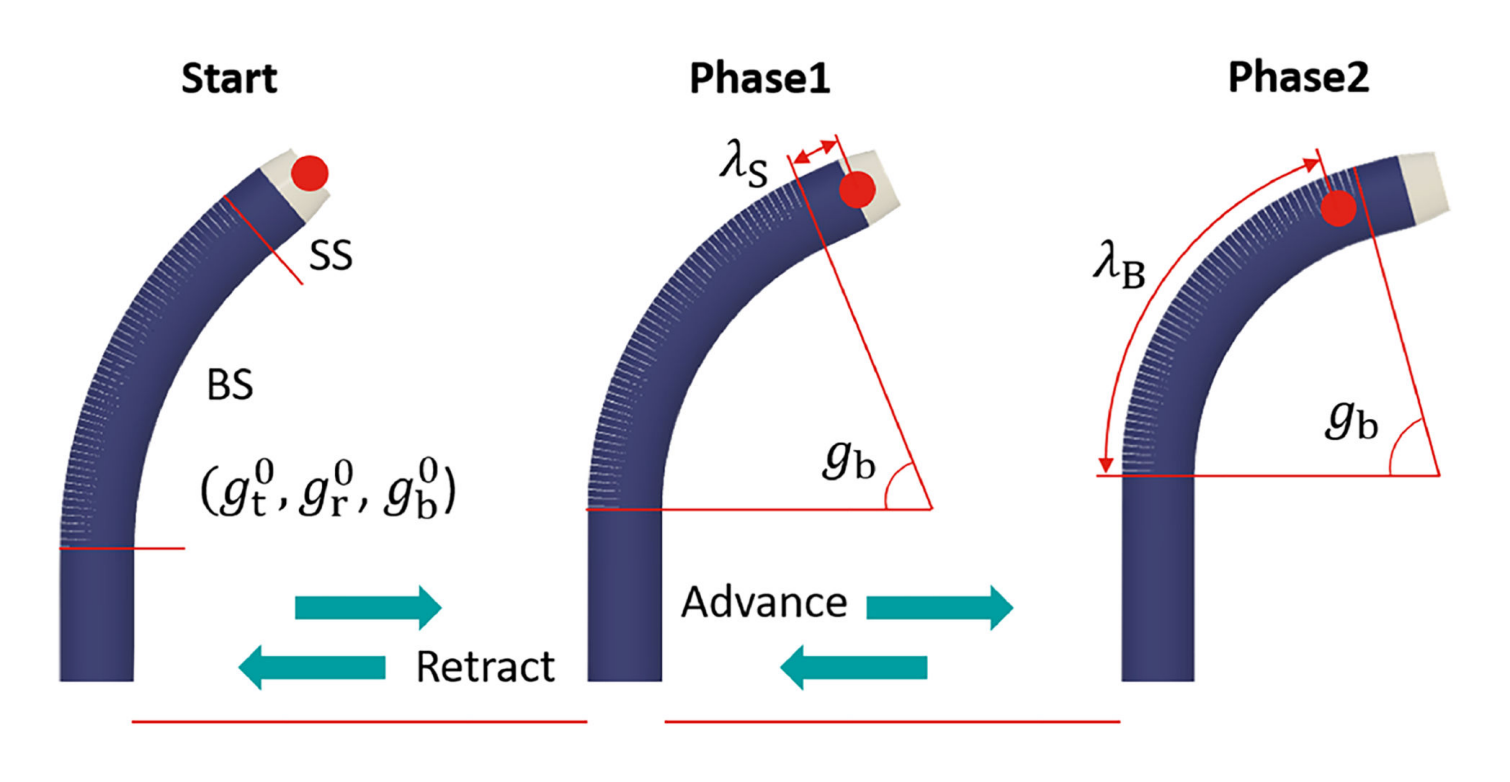
Point-constrained control comprises two phases: firstly, crossing the SS, followed by traversing the BS.

To achieve this delivery, a fixed point is initially established at the tip of the SS (the red dot as shown in Fig. 5), with the configuration parameters recorded as $\left(g_{\text{t}}^{0},g_{\text{r}}^{0},g_{\text{b}}^{0}\right)$. Its position is computed as ${\bar{S}}^{0}={\bar{S}}_{\text{S}}\left(G_{\text{S}}^{0}\right)={\left[{\bar{X}}_{\text{S}}^{0}{\bar{Y}}_{\text{S}}^{0}{\bar{Z}}_{\text{S}}^{0}\right]}^{T}$. The control objective is then simplified to compensate for the bending angle required to ensure the shape passes through this point while translating the catheter within the x-z plane of $\left\{{\bar{\text{F}}}^{\text{p}}\right\}$. It turned

Algorithm 1 **Point-Constrained Control.**

**Input:**$G_{\text{S}}^{0}$, $g_{\text{t}}^{e}$, Phase

**Output:** *U*

1: ${\bar{S}}^{0}\leftarrow {\bar{S}}_{\text{S}}\left(G_{\text{S}}^{0}\right)$ ⊳(15)

3: **while** within robot limits **do**

4:         **if** advancing **then** $g_{\text{t}}^{e}\leftarrow g_{\text{t}}^{e}+0.1$

5:         **else if** retracting **then** $g_{\text{t}}^{e}\leftarrow g_{\text{t}}^{e}-0.1$

6:         **end if**

7:         **if** Phase == 1 **then**

8:           $U\leftarrow f_{\text{S}}^{-1}\left(\lambda ,g_{\text{b}},g_{\text{t}}^{e}\right)$ ⊳ (16)

9:           **if** λ == 0 **then** Phase *←*2

10:          **end if**

11:         **else if** Phase == 2 **then**

12:           $U\leftarrow f_{\text{B}}^{-1}\left(\lambda ,g_{\text{b}},g_{\text{t}}^{e}\right)$ ⊳ (17)

13:           **if** λ == *g*_b_ **then** Phase*←* 1

14:           **end if**

15:         **end if**

16:         **return***U*

17: **end** **while**

out to be the solution of non-linear equations with an expected translational trajectory $g_{\text{t}}^{e}$. This comprises two phases: firstly, crossing the SS as outlined in (16), followed by traversing the BS as specified in (17).

(16) $$f_{\text{S}}\left(\lambda_{\text{S}},g_{\text{b}},g_{\text{t}}^{e}\right)={\bar{S}}_{\text{S}}\left(\lambda_{\text{S}},g_{\text{t}}^{e},g_{\text{r}}^{0},g_{\text{b}}\right)-{\bar{S}}^{0}=0_{3\times 1}$$

(17) $$f_{\text{B}}\left(\lambda_{\text{B}},g_{\text{b}},g_{\text{t}}^{e}\right)={\bar{S}}_{\text{B}}\left(\lambda_{\text{B}},g_{\text{t}}^{e},g_{\text{r}}^{0},g_{\text{b}}\right)-{\bar{S}}^{0}=0_{3\times 1}$$

where *f* : ℝ^2^ → ℝ^3^ is the nonlinear system and *U* = (*λ, g*_b_) is the solution vector of it. The whole algorithm is summarized in Algorithm 1.

## III Experiments

In this section, we aim to evaluate the performance of the robotic system, focusing on control accuracy and feasibility. Initially, we manufactured the robotic system and integrated it with an NDI Aurora system (Waterloo, Ontario, Canada) and a camera setup to measure the accuracy in both tip position control and point-constrained control. Subsequently, we designed and fabricated a phantom model to validate the ability of the SDS to navigate clinically relevant trajectories and to assess its effectiveness in improving navigation efficiency and reducing vessel wall contact force.

### A Control Accuracy Experiments

**Fig. 6:**
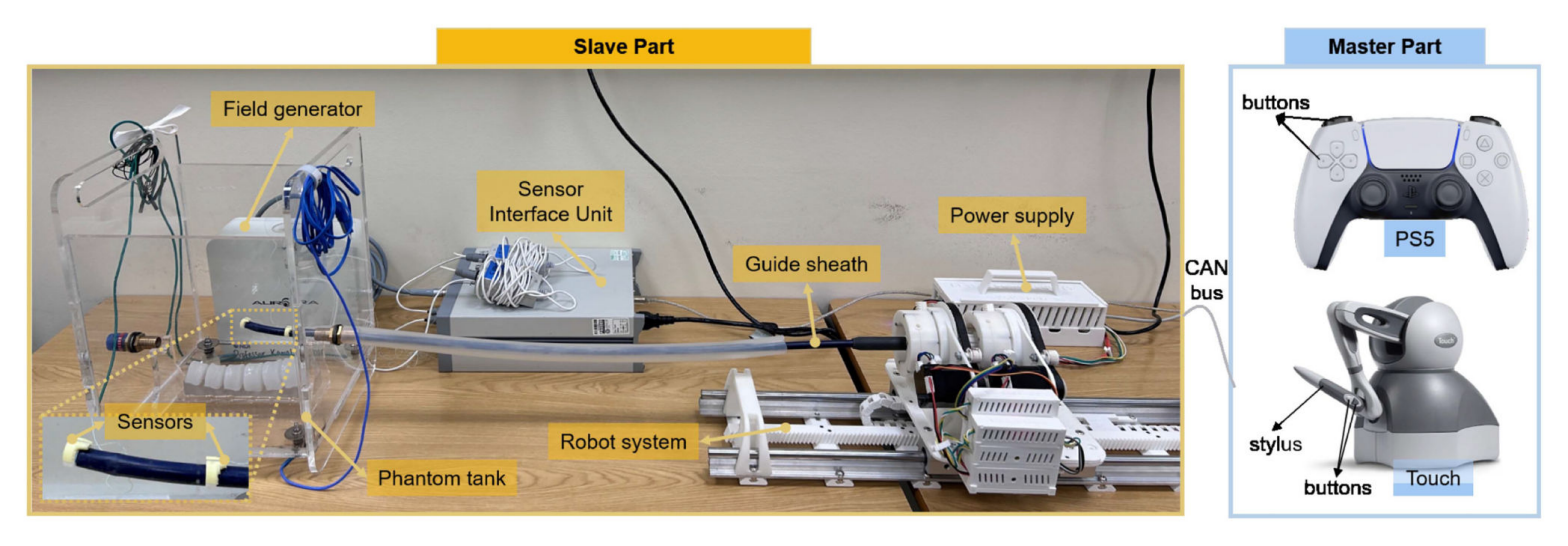
Experimental setup for control performance evaluation: The master part includes two controllers (PS5 and Touch device). The slave part (robot) integrates a phantom tank and an NDI Aurora system for tracking tip poses. They are connected via a 25-meter-long cable and communicate using CAN protocol.

The proposed tip position and point-constrained control algorithms were implemented and evaluated on the robotic system capable of teleoperating sheaths based on a master-slave configuration (Fig. 6). A Geomagic Touch haptic device (”Touch”, 3D Systems, Rock Hill, South Carolina, USA) is utilized as the master controller, with its stylus tip providing 3D pose data for tip position control. The device’s two buttons enable point-constrained control by advancing or retracting the sheath. Additionally, a PlayStation 5 dualsense wireless controller (PS5, Sony, Minato, Tokyo, Japan) is employed for joint-level control, with each button corresponding to a specific joint. Both controllers function solely as input devices and can be replaced by other devices with similar functionality. Control commands, generated by the PS5 or Touch, are transmitted via a 25-m-long communication cable to the slave part.

In our simulation setup, we utilized an acrylic tank containing a doped spine (enhanced with radiopaque materials) to emulate the anatomical environment resembling a heart. Within this setup, brass inlets positioned on the superior and inferior anatomical walls respectively represented access points for the superior vena cava (SVC) and IVC. The robot can manipulate standard guide sheaths (Edwards Lifesciences, Irvine, CA, USA) in 3 DoFs, with dimensions listed in Table I. The guide sheath BSs measure 50 mm and 42 mm in length and have outer diameters of 30 Fr and 22 Fr, respectively. For other standard guide sheaths with different lengths, a similar manipulation performance is expected, since the structures are similar.

#### 1) Tip Position Control Experiment

To comprehensively assess tip position control performance, we conducted three tests. The first evaluated accuracy using a predefined figure eight (“8”) trajectory (40 × 20 mm^2^), comprising straight lines along the left to assess precise linear control and orientation transitions, and a curved path to evaluate the ability to track nonlinear trajectories smoothly. The combination of straight and curved segments reflects common motion patterns in real-world applications, such as catheter navigation through vascular pathways. The second test simulated real-world manipulation by examining responses to random user inputs using the ‘Touch’ device, introducing variability in curvatures. We synchronized the movements of the stylus tip with the sheath tip’s expected position when the button of “Touch” was pressed. The third test, simulating X-ray-guided situation, involved random manipulation in 2D space, exemplified by (LAO 31°, CRA 34°) and (RAO 29°, CAU 43°). To ensure safety, the sheath tip was constrained to stop following when crossing the boundaries of the robot workspace. These tests reflect clinical scenarios, offering a comprehensive evaluation.

To measure the performance, 6-DoF electromagnetic (EM) sensors, a sensor interface unit and a field generator (NDI Aurora, Waterloo, Ontario, Canada) were employed to track the tip poses. Customized connectors were designed to connect two EM sensors with the proximal end of the BS and the tip. The tip pose was derived from the transformation matrix between the two sensors. Error calculations were performed by first aligning the initial real-time tip position with the corresponding expected position. Subsequently, the relative distance between the realtime tip positions and the expected positions was measured to assess accuracy.

#### 2) Point-Constrained Control Experiment

To further investigate the performance of the control strategy proposed in Section II-D, point-constrained delivery experiments were conducted. Two buttons on the “Touch” stylus were employed to control the advancing and retracting of the sheath until it reached its limits. Then the shape of the tip would adjust automatically through bending based on the point-constrained control algorithm. The sheath’s curvature can vary after determining the fixed point, leading to different initial bending before applying point-constrained control. Therefore, a comparison between noload and half-load bending conditions was conducted to evaluate the impact of initial curvature on performance.

**Fig. 7:**
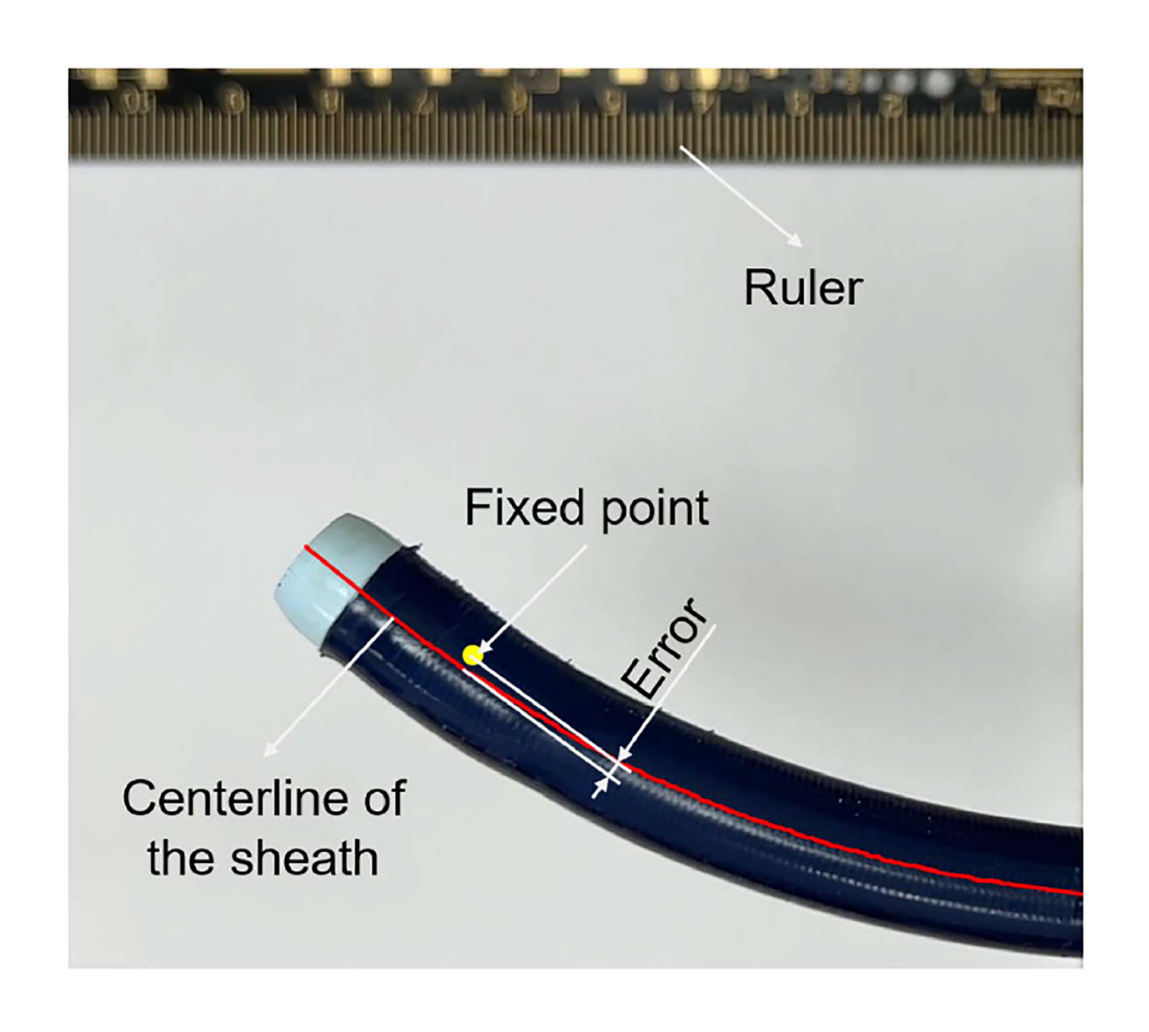
Accuracy measurement process of point-constrained control.

The accuracy was evaluated by measuring the shortest distance between the fixed point and the centerline of the BS during the delivery, as shown in Fig. 7. A video with a resolution of 1920 × 1080 pixels and a rate of 30 frames/s was recorded using a camera (iPhone 14 camera, Lens correction) positioned at a height of 28 cm. The fixed point was chosen based on the structural features of the BS shown in the initial frame. The sheath centerline was reconstructed using a skeletonization algorithm based on [24]. Initially, each frame was converted to the Lab color space to improve color segmentation, with a focus on extracting the ’b’ channel (blue-yellow channel). Subsequently, a mask was created to isolate the catheter color, and the inverted mask was used to obtain the catheter outline. Any internal holes within the catheter outline were filled, and isolated areas were connected to ensure continuity. Finally, skeletonization was applied to extract the centerline, which was further refined using a regression spline. Additionally, a ruler positioned at a height of 0 cm facilitated the calculation of pixel width. Subsequently, similar triangles were employed to determine the pixel length of the sheath at a height of 19cm. Then, the distance in pixels was converted to an error in millimetres to assess the accuracy of the control method. Additionally, we employed the sheath diameter as a metric to validate the reasonableness of the error. If the fixed point lies within the sheath, the error should be less than half of the diameter. Considering random error associated with centerline extraction and fixed point selection, we conducted advancing and retracting experiments five times.

### B Feasibility Experiments

There are two objectives for the experiment: a) to validate the SDS’s feasibility along clinically relevant trajectories and a) to assess its effectiveness in enhancing navigation efficiency and minimizing tissue wall contact forces through comparative analysis with conventional robotic JC and MC.

#### 3) Experimental Setup

**Fig. 8:**
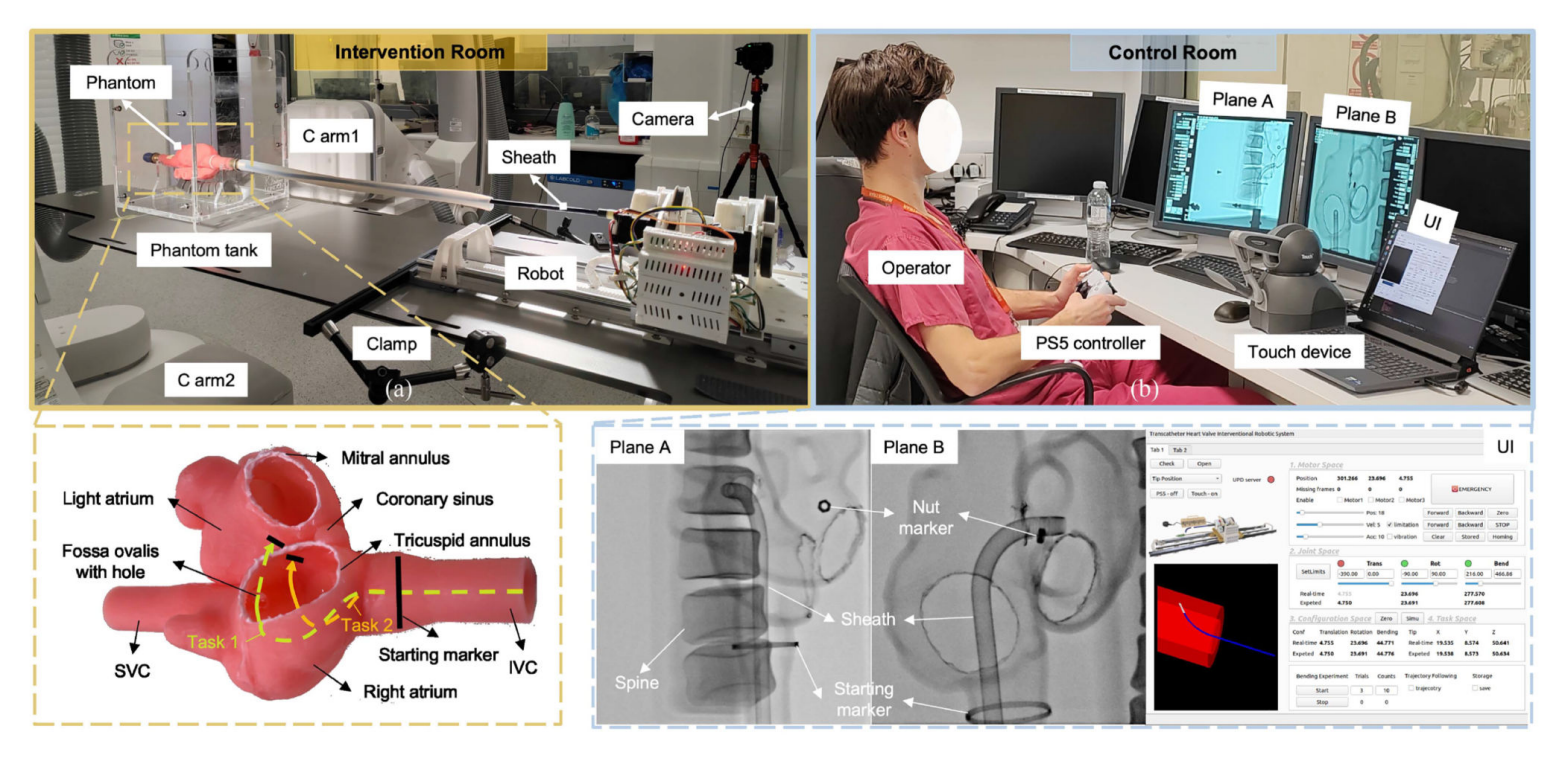
Setup for feasibility experiments: (a) Intervention room: Placement of the robotic system within the intervention room, with the heart phantom displaying anatomical structures for two tasks: crossing the septum and approaching the TV annulus. (b) Control room: Bi-plane imaging and UI guidance for navigation.

The setup is illustrated in Fig. 8. The robot was securely mounted to the patient bed using clamps in the intervention room (Fig. 8(a)) and interfaced with a phantom tank housing the heart model. To replicate clinical scenarios, we focused on example procedural objectives during cardiac transcatheter interventions (including MV and TV interventions, and specifically sheath delivery). Subsequent steps, such as catheter deployment and valve repair or replacement, were not included. Therefore, the developed heart model only contains key anatomical structures necessary for sheath navigation: left atrium, right atrium, IVC, Eustachian ridge, SVC, radiopaque mitral annulus, and radiopaque tricuspid annulus, with a deliberate 12 mm radiopaque aperture in the septum located on the fossa ovalis. Ventricles and valves were intentionally omitted for simplification. Derived from a patient CT scan, the model’s geometry and complexity were validated in our previous work [25], demonstrating sufficient accuracy for guiding catheters and performing electroanatomical mapping, with performance comparable to leading interventional simulators. Constructed from F80 Resin (RESIONE, Dongguan, China) with 1:1 real dimensions, it features a soft texture, mimicking cardiac tissue properties. The radiopaque resin was affixed to two annuluses and the fossa ovalis to enable visualization under X-ray. While completion times are expected to be shorter than in actual procedures, crossing the fossa ovalis and centring the sheath at the TV annulus remain challenging, requiring refined manipulation skills.

Two sheath delivery steps were selected for validation: a) Task 1 - Navigate from the starting marker and cross the fossa ovalis in the septum by 20 mm; b) Task 2 - Navigate from the starting marker to the centre of TV annulus and cross the annulus by 20 mm. These paths represent the characteristic challenges and complexities encountered during interventions, including navigating through the fossa ovalis and the tight curves typically encountered in transfemoral tricuspid procedures.

#### 2) Experimental Conditions

The evaluation began with an introduction to the objectives and setup in the control room (Fig. 8(b)), followed by a 20-minute training session conducted in free space (without the phantom) under fluoroscopic guidance to familiarize the operator with the JC (using PS5) and SDS (using Touch) methods. The catheter and phantom were visualized on the fluoroscopy screen. This training covered the technical operation of these devices, including the use of six buttons on the PS5 to control the three DoFs independently. Additionally, the operator learned to use the two buttons on the Touch device to manage tip position control (start/stop) and point-constrained control (advance/retract). For MC, the operator stood in the intervention room, while for JC or SDS, the operator sat in the control room and manipulated remotely using a PS5 or Touch device. Bi-plane fluoroscopy was used to guide the procedure, maintaining consistent X-ray imaging angles (Plane A: RAO 30°, CAU 0°; Plane B: LAO 65°, CAU 6°) across trials to eliminate the influence of view variation on the results. An additional nut marker attached to the phantom was used to track its movement. The UI allowed the operator to monitor key robot information, including positions, limits, and other relevant parameters, during manipulation.

#### 3) Participants

Three structural cardiology fellows from St. Thomas Hospital participated in this study. Two had 1–3 years of experience, while one had 5-6 years of experience. No individual had extensive prior experience with the robotic system and no test runs took place before the execution of the experiments. To ensure trial independence, participants arrived at separate times and were not influenced by one another. Each operator performed two predefined tasks using two control methods (JC and SDS), with each task repeated twice, resulting in 36 data sets (3 operators × 2 tasks × 2 methods × 3 repetitions). Manual control was used as the ground truth for comparison. Additionally, the experienced cardiologist performed each task five times, generating 10 data sets (2 tasks ×5 repetitions).

#### 4) Evaluation Metrics

All images were acquired using an Artis Q bi-plane angiography unit (Siemens Healthcare GmbH, Forchheim, Germany) and transferred to a personal computer at 30 frames/s via a Razer Ripsaw HD frame grabber (Razer Inc., Irvine, California, USA). After the study, a calibration cube was placed near the phantom to ensure clear visualization of all markers. X-ray images of the cube were captured under the same views as the phantom. Positional data for the sheath tip (*P*_tip_) and phantom (*P*_phantom_) were extracted using SARA (System for Angiographic Reconstruction and Analysis) software [26]. Statistical analyses were performed using R software, with performance differences between SDS and MC or JC evaluated via the Mann–Whitney U test. Statistical significance was set as p < 0.05. Results are presented as box plots. The metrics for evaluating navigation efficiency are detailed below. Optimal performance was defined by shorter OT, reduced TL, less RJ, shorter DT, less ID (contact force), higher SR, and smaller DS.

*a) Operation time (OT):* This was measured as the duration (in seconds) from the starting marker to task completion using a stopwatch.

*b) Trajectory length (TL):* This represents the sum of Euclidean distances between consecutive points along the recorded path:

(18) $$\text{TL}=\sum_{i=1}^{n-1}||P_{\text{tip}}^{i+1}-P_{\text{tip}}^{i}||$$

where *n* is the total number of recorded points.

*c) Root mean square of the jerk (RJ):* This metric quantifies the smoothness of motion, with jerk representing the rate of change of acceleration. It is calculated as the square root of the mean of the squared jerk values across all three dimensions for the entire trajectory, given by:

(19) $$\text{RJ}=\sqrt{\frac{1}{n-3}\sum_{i=1}^{n-3}||\frac{P_{\text{tip}}^{i+3}-3P_{\text{tip}}^{i+2}+3P_{\text{tip}}^{i+1}-P_{\text{tip}}^{i}}{{\left(\text{Δ}t\right)}^{3}}|{|}^{2}}$$

where Δ*t* is the time step between consecutive points.

*d) Dwell time (DT):* This refers to the duration that the system spends in a specified region of interest or near a target, with a velocity threshold of 1 mm/s. This includes not only the time spent at the target location but also any fine adjustments made to ensure precise positioning.

(20) $$\text{DT}=\sum_{i=1}^{n-1}\text{Δ}t(i)\;\text{ where }\;||\frac{P_{\text{tip }}^{i+1}-P_{\text{tip }}^{i}}{\text{Δ}t}||<1$$

*e) Sheath-induced displacement (ID) and successful retraction rate (SR):* For Task 1, an additional metric was tissue contact force, indirectly assessed by monitoring the movement of the non-fixed phantom tank. Retraction from the fossa ovalis was deemed a failure if ID exceeded 25 mm. The success retraction rate was used as the performance metric for this criterion.

(21) $$\text{ID}=\sum_{i=1}^{n-1}||P_{\text{phantom }}^{i+1}-P_{\text{phantom }}^{i}||$$

*f) Distance to TV annulus center (DS):* For Task 2, accuracy was evaluated by measuring the distance between the center of the TV annulus and the centerline of the catheter in the x-ray images with SARA.

## IV Results and Discussion

### A Tip Position Control Performance

**Fig. 9:**
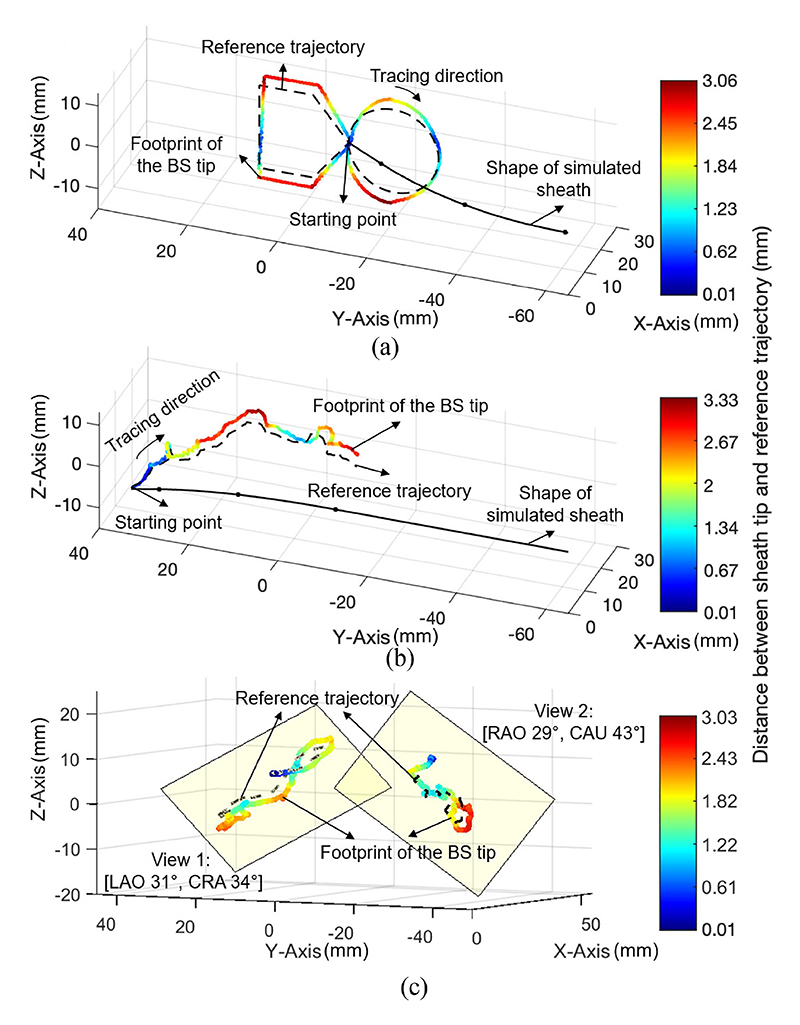
Tip trajectory following results of the large sheath. (a) “8” shape following with an RMSE of 1.96 mm. (b) 3D trajectory following using the Touch device with an RMSE of 2.10 mm. (c) 2D trajectory following using the Touch device: view 1 with an RMSE of 2.00 mm and view 2 with an RMSE of 2.11 mm.

The diagram in Fig. 9 shows the tip following results of the large guide sheath. Dashed lines represent the reference trajectories, either “8” or “Touch” stylus tip positions. The shape of the simulated sheath reflects the initial status of the trial, and the tracing direction indicates its subsequent movement. The sheath tip footprints were recorded by the EM tracking system. The error from the tip to the reference trajectories is indicated by the warm color gradient. In Fig. 9(a), the “8” trajectory following process started at the centre point. The error starts small upon initiating the stylus tip tracking. However, it noticeably increases while moving along the positive and negative directions of the Z axis, with a maximum error (Max) of 3.06 mm. Despite this, most parts of the trajectory tracking are smooth and closely follow the reference, with the trends remaining consistent. Both master 3D (Fig. 9(b)) and 2D (Fig. 9(c)) following demonstrate similar results, with Max values of 3.33 mm and 3.03 mm, respectively. This increase in error is primarily attributed to twist and bend observed in the sheath’s long shaft. Simultaneously, we simplified the sheath’s bending model, without incorporating twisting and shear strain, to achieve relatively real-time control but increase tip errors.

Root mean square error (RMSE), standard deviation (SD), and Max of the positioning results are summarized in the first four lines of Table II. For the large sheath trials, the RMSE averages 2.04 mm, with an SD of 0.85 mm. In comparison, the small sheath exhibits a higher overall error, with the RMSE averaging 2.15 mm and an SD of 0.89 mm. This is because of the less accurate kinematic model from the prior work [23]. In real situations, larger heart sizes are likely to increase tip position error, particularly along the Z-axis.

### B Point-Constrained Control Performance

**Fig. 10:**
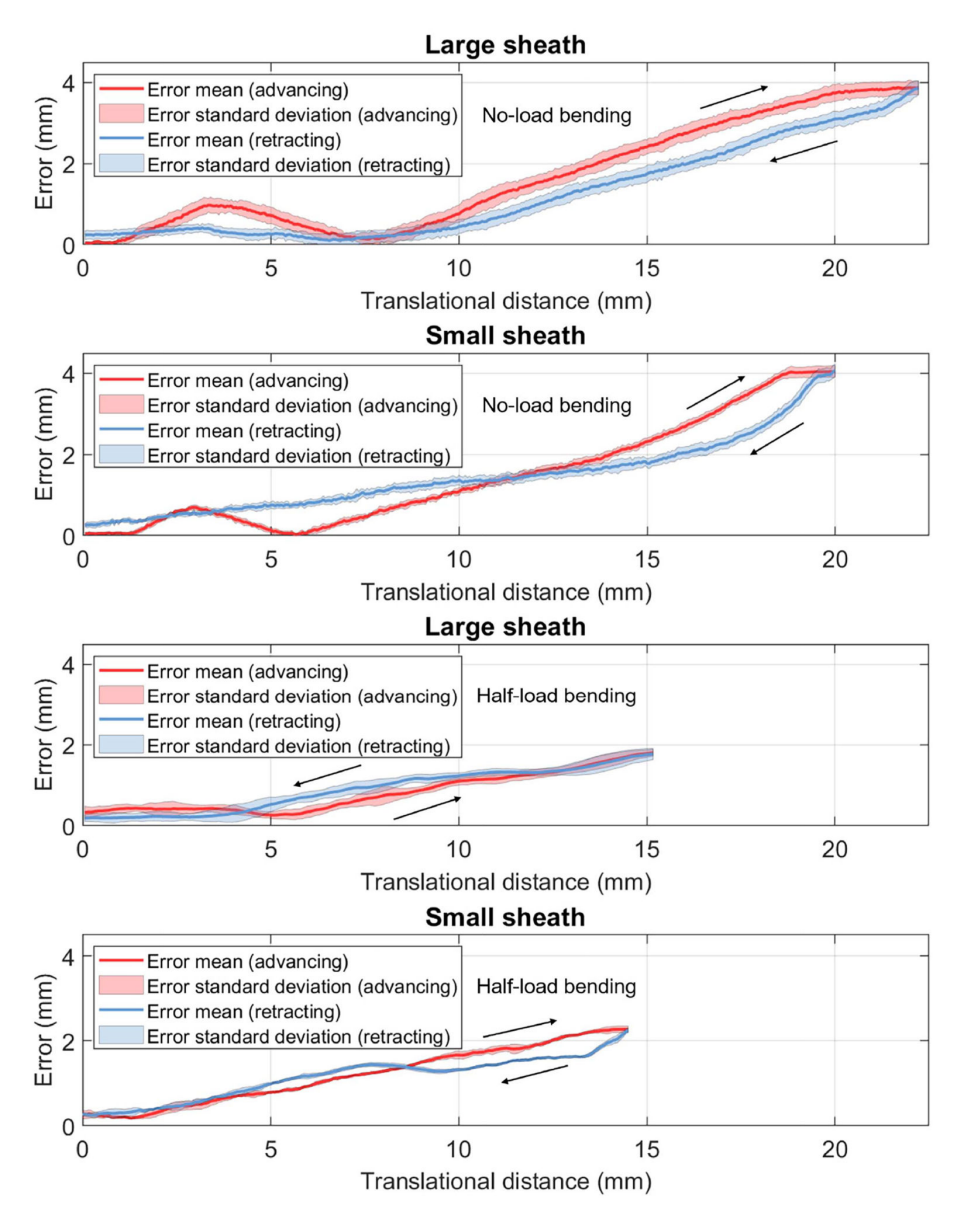
Point-constrained delivery results of two sheaths with two load conditions: The large sheath achieved a Max of 3.88 mm. The small sheath achieved a Max of 4.06 mm. The overall standard deviation of the error in the small sheath is smaller than in the large sheath.

The errors observed during the advancement and retraction of the sheaths are illustrated in Fig. 10. Notably, the error was near zero at the beginning because the fixed point was on the centerline. During no-load bending advancement, the error initially increases slightly, followed by a decrease and subsequent increase. This variation is attributed to transmission latency within the cable-driven bending mechanism, leading to delayed wire tensioning. As the sheath moves forward (to the left as shown in Fig. 7), insufficient bending movement occurs, causing the fixed point to shift relative to the right side of the sheath, thus increasing the error. Subsequently, the tension in the sheath shaft results in greater bending than expected, causing the fixed point to shift slightly from the right side to the left side of the sheath. This phenomenon explains the observed pattern of decreasing and then increasing error. At its peak, the Max reaches 3.88 mm. The RMSE and SD values of the mean error line are 2.12 and 1.31 mm, listed in Table II. During retraction, the tension in both the tip and the long shaft is smoothly released. Consequently, their relative locations converge, leading to a reduction in error. As the structure returns to its original form, the slope of the curve in the figure increases compared to the advancing stage. The presence of minor slack in the gear rack translational mechanism, along with the prolonged recovery process due to residual tension, results in a 0.23 mm offset at the end. Similarly, the small sheath exhibits analogous trends during advancement but with a larger Max of 4.06 mm, due to its relatively less precise kinematic model. When comparing the retracting process, the tension release in the small sheath is initially rapid, followed by a slower decline. Notably, the overall SD of the error (enclosed areas in the graph) in the small sheath is smaller than in the large sheath, likely due to the larger shape introducing more variability during centerline extraction. The RMSE and SD of the mean error line for the small sheath are 1.94 mm and 1.27 mm, respectively.

Compared to the no-load bending condition, the translational distances of the half-bending condition are shorter in both catheters due to bending limitations, with reduced Max: 1.80 mm for the large sheath and 2.28 mm for the small sheath. The pre-existing tension in the catheter bypasses the tension-loading phase, leading to a steady initial increase in errors and lower RM-SEs: approximately 1.02 mm for the large sheath and 1.34 mm for the small sheath (the worst case results from advancing and retracting). Beyond this phase, the error curves closely align with those observed in the no-load condition.

The average RMSEs (the first six lines in Table II) for tip position and point-constrained control are 2.10 mm and 1.86 mm, which are within clinically acceptable limits, considering the heart’s movement during the cardiac cycle. Additionally, clinicians routinely assess device deployment using angiography and can make adjustments if necessary.

### C Feasibility Performance

All 46 trials were successfully completed, with all trials using SDS achieving successful retraction. These results demonstrate that SDS is a feasible method for sheath delivery in a phantom setting, effectively aligning with the TV annulus, crossing the fossa ovalis, and ensuring reliable retraction.

**Fig. 11:**
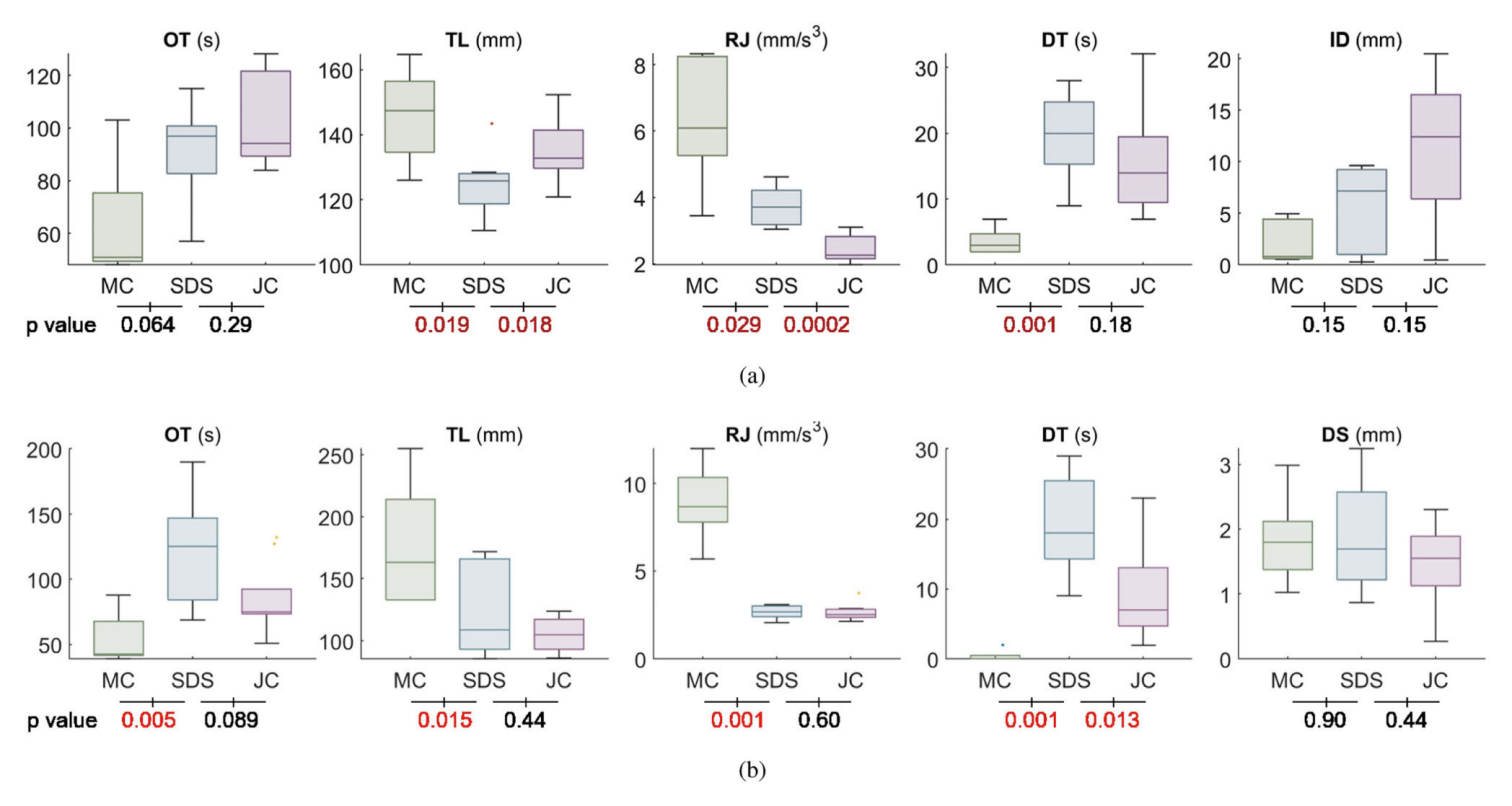
Box plots of the results, including OT, TL, RJ, DT, ID, and DS, with p-values between SDS and MC or JC listed below the plots. Significant p-values are marked in red. (a) Results for Task 1 and (b) Results for Task 2.

The results are presented in Fig. 11, with p-values displayed below the corresponding plots. Significant differences are high-lighted in red. In Task 1, SDS required a longer OT (median: 97 s) and DT (median: 20 s) compared to MC (OT median: 51 s; DT median: 3 s). However, it achieved significantly lower TL (median: 125.73 mm; p = 0.019) and RJ (median: 3.72 mm/s^3^; p = 0.029) compared to MC (TL median: 147.37 mm; RJ median: 6.08 mm/s^3^). While manual trials were quicker and involved less hesitation due to the cardiologist’s familiarity with the procedure, SDS reduced unwanted movements and resulted in smoother operations. For ID, MC achieved a median of 0.82 mm with a 100% SR due to the cardiologist’s ability to sense and adjust force during retraction. In contrast, JC resulted in a median ID of 12.44 mm and an SR of only 44.44%, reflecting the challenge of balancing translation and bending during retraction. SDS improved performance with a median ID of 7.14 mm and a 100% SR, enabled by point-constrained control, which allows the user to fix a point and deliver the sheath steadily by pressing a button. Although JC caused less RJ (median: 2.26 mm/s^3^) due to small adjustments between translation and bending, it required longer TL (median: 132.9 mm; p = 0.018). SDS’s higher DT is attributed to the need for switching between position control in different views.

In Task 2, the comparison between SDS and MC follows a similar pattern to Task 1. While no significant differences in DS were observed, SDS exhibited greater variability (IQR: 1.36 mm). The variations in SDS performance are attributed to the increased operator freedom in handling the stylus within the larger available space, which allows more flexible movement. This, in turn, contributed to larger variations in OT (IQR: 63.25 s) and TL (IQR: 72.74 mm) compared to JC. In contrast, JC showed better performance in OT (median: 75 s), DT (median: 7 s), as it benefited from the absence of spatial constraints. In summary, these findings demonstrate that SDS reduces contact force compared to JC, enabling safe and effective retraction from the fossa ovalis with robotic assistance. It also results in fewer unwanted movements and smoother operations compared to MC. However, in larger free spaces, SDS requires more caution and adjustments to maintain optimal performance.

When applying in actual patient scenarios, there are four main differences that should be considered: 1) The phantom lacks the right and left ventricles. While this does not impact the current test, cardiologists in real cases must navigate carefully to avoid interactions with structures such as the chordae tendineae; 2) The phantom features radiopaque markers to improve visibility during fluoroscopy, while in live procedures, soft tissues and bone structures can obstruct the view, making both manual and robotic navigation more challenging; 3) Blood flow affects catheter positioning, requiring cardiologists in real cases to adjust catheters to minimize this effect; and 4) The phantom model remains static, whereas in real patients, cardiac and respiratory motions introduce slight positional shifts that impact catheter navigation. The absence of these motions in the phantom limits the realism of the simulation. Despite these differences, the phantom model provides a controlled environment to evaluate the feasibility of the robotic system and compare results across different control methods. The findings demonstrate performance outcomes comparable to typical clinical procedures. However, further development, including addressing motion effects as well as adequate operator training, are essential for the smooth integration of this system into real-world clinical settings.

## V Conclusion And Future Work

This paper presents the development and evaluation of a 3-DoF robotic system to enhance the safety and efficiency of cardiac sheath delivery through challenging anatomical structures. The system incorporates a novel SDS with tip position control for precise entry navigation and point-constrained control for shape-constrained crossing. Technical evaluations showed average RMSEs of 2.10 mm for tip position control and 1.86 mm for point-constrained control. Feasibility experiments with a validated phantom model demonstrated that SDS reduced tissue wall contact compared to JC and provided smoother, more controlled operations than MC, ensuring safer and more effective delivery through confined pathways. Notably, SDS requires careful adjustments when operating in larger free spaces. This work advances robotic-assisted cardiac interventions, offering a reliable and safer method for navigating complex anatomical structures.

Future efforts will focus on developing a hybrid strategy that combines JC for rapid navigation with SDS for precise adjustments. Additionally, a comprehensive user study will be conducted to validate system performance further. Plans also include integrating multiple catheters to facilitate device deployment in confined anatomical spaces.

## Figures and Tables

**TABLE I:** Two Experimental Sheaths From Edwards Lifesciences

	BS dimension		Apperance	
	L (mm)	D (Fr)	Tip	Handle
Large sheath	50	30	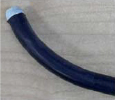	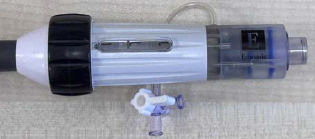
Small sheath	42	22	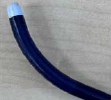	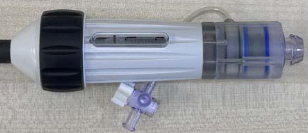

L - length; D - diameter.

**Table II:** Bs Tip Position Rmse, Sd, And Max Of Two Sheaths

	Large sheath			Small sheath		
	RMSE (mm)	SD (mm)	Max (mm)	RMSE (mm)	SD (mm)	Max (mm)
”8” trajectory following	1.96	0.79	3.06	2.45	0.95	4.42
Master 3D following	2.10	0.88	3.33	2.37	0.94	4.11
Master 2D following (view 1)	2.00	0.85	3.03	1.93	0.82	3.45
Master 2D following (view 2)	2.11	0.86	3.67	1.86	0.86	3.41
Constrained advancing (no-load bending)	2.12	1.31	3.88	1.94	1.27	4.05
Constrained retracting (no-load bending)	1.70	1.14	3.87	1.67	0.89	4.06
Constrained advancing (half-load bending)	0.94	0.47	1.80	1.34	0.67	2.28
Constrained retracting (half-load bending)	1.02	0.51	1.77	1.22	0.51	2.25
